# Points from Hospital Reports

**Published:** 1924-08

**Authors:** 


### POINTS FROM HOSPITAL REPORTS.

### St. George's Hospital.

The year 1923 was a busy one in all departments, the total number of patients, both in and out, being greater than in 1922. The one unsatisfactory feature of the year was the disparity between income and expenditure. Although there was practically no extraordinary expenditure, the total spent exceeded the income by a sum but a few pounds short of £16,000, and when we add that in 1922 the amount of the adverse balance exceeded £18,000, the gravity of the financial position of this great hospital will be but too evident. Two special incidents marked the year—co-operation with the Hospital Saving Association, and the establishment of a maternity unit. The Report informs us that from the former the hospital benefits materially, "as in many cases, where little or no contribution would be received from an in-patient, the Association pays a large part of the cost of maintenance, and in the case of out-patients contributes the actual average cost per patient of treatment." A cursory scanning of the accounts does not reveal what the amount of the contributions of the Association was. The maternity unit, which was formed on the recommendation of the Medical School Committee, consists of 11 beds and 11 cots, the Wellington Ward, previously for women surgical patients, having been converted to this purpose. It was opened in May, and in the seven months 155 babies were born in the unit. An extension of the out-patients' department, involving an expenditure of £16,500, towards which the King's Fund has made a grant of £500, is about to be taken in hand

### National Hospital for the Paralysed and Epileptic.

On the whole the year 1923 was an uneventful though prosperous one for the National Hospital in Queen Square, marked by steady work, the maintenance of income generally, and the improvement of several of the more vital sources thereof, and the effective control of expenditure. The account of income and expenditure shows a balance on the right side at the end of the year of £1,751, which, with a pre-existing balance of £5,810, gives a total unexpended balance on that account of £7,561. It is thus evident that the hospital is to be congratulated on having extricated itself from the serious financial difficulties by which it was recently beset. The Board can therefore now devote attention to necessary improvements, and are accordingly engaged in building an urgently needed enlargement of the electrical department at a cost of £3,525. The expansion and alphabetical arrangement of the index would give the finishing touch to this otherwise excellent report.

### Cardiff Royal Infirmary.

This hospital of 310 beds, formerly known as King Edward VII. Hospital, Cardiff, will henceforth bear the above title. The change follows the recent incorporation of the hospital by Royal Charter. The year 1923 was marked by increased work, both among in-patients and out-patients, by further reduction in expenditure, and by a material development of the income, which, for the first time for several years, showed a balance on the right side. The Report is an admirable document, excellently printed, and gives, not only all the desirable statistics for the year, but, side by side with them in tabular form, those of past years as well, which adds so much to the information and interest derivable from such publications.

### Norfolk and Norwich Hospital.

The year 1923 was full of work, accompanied by material reduction, extending over practically the whole field. The valuable statietics of which we have just spoken, for example, are not included in it; while the income, although exhibiting under some important heads a falling off not very material in amount, displays, on the other hand, some very satisfactory features. Chief among these is the still further development of the large sum provided by the Hospital Sunday and Hospital Saturday Funds (£18,486), the bulk of which is furnished by the contributory scheme, which, furthermore, provided during the year £1,250 for the installation of an electric lift, a photograph of which appeared in our June number. This is one of many provincial hospitals which have adopted the model form of income and expenditure account of the Uniform System of Hospital Accounts, without the statistical tables which are its essential appendages. Useful as is the former in any case, since it ensures identity of projection and the inclusion under each particular heading of precisely the same things, it is shorn of some of its value by the absence of the statistics of work and of average cost of in-patients and out-patients respectively under the eight classes of expenditure, which these tables are framed to supply. We would strongly recommend their inclusion in the annual report of every hospital which has adopted the Uniform System. Speaking of the income and expenditure account, we observe two peculiarities in this case—that in 1922 £1,446 was written off donations and transferred to the balance-sheet for investment, and that in 1923 the gross receipts on account of private nurses were written off maintenance salaries and wages. The Uniform System in its integrity would have shown these figures in each case on the other side of the account and presented the full figures of donations and salaries and wages respectively. We also suggest that the inclusion of a separate laundry account on the lines of the Uniform System is worth considering. It would show the full cost of washing, not now deducible from the accounts.

### Staffordshire General Infirmary.

For this hospital the year 1923 was characterised by steady work and was unmarked by any event of outstanding prominence. All that it is necessary to say of its operations may therefore be said in a few words. Happily the relation is a very satisfactory one—a full year's work, expenditure still further materially reduced, and income sufficient, within £254, to defray it. In looking through the Report the number of beds available has not caught our eye; we suggest that it might be added to the comparative statement on page 16. Our search for the index has also been ineffectual. We think no report should be without such a guide, especially when there is so much of interest in it as in the one before us.

### Belgrave Hospital for Children.

That this is the Children's Hospital for London South of the Thames is effectively demonstrated by the fact that in 1923 1,448 of its 1,496 in-patients were South Londoners. It contains 50 beds, of which 44.6 were in daily occupation, a high percentage. The number of in-patients was 344 higher than in 1922, while out-patients were rather less numerous. Expenditure was well under control, and this, coupled with the increased number of in-patients, improved the average daily cost per head. It is pleasant to see such splendid work in the less opulent part of London being successfully financed, as it was last year. It requires constant endeavour, and we congratulate the Committee and their Secretary, Mr. Clapham, on the enterprise which they bring to their labour of love.

### Newton Hospital, Massachusetts, U.S.A.

Newton is a "city" of some 50,000 inhabitants. We cannot discover the number of beds in the hospital—a piece of information about which the Reports of many hospitals nearer home are also strangely reticent—but the average number occupied daily throughout the year was 105, which is exactly the same figure as that of the Hampstead General Hospital for 1922. This gives us an idea of the size of the institution, but there the analogy ends. Newton Hospital is, to a large extent, a pay hospital. In the year under review, ending August 31, 1923, only 21 per cent. of its patients were free, 42 per cent. contributed part of the cost of treatment, and 37 per cent. more than cost. This is very interesting information, and now that patients' payments have become so considerable a source of income to hospitals in this country, we think every Report should contain a similar statement. The total expenditure was $180,000, and the earnings $145,500. Of the balance of $34,500, $18,000 was met by interest on investments, leaving only $16,500 to be provided by voluntary contributions, and as that source yielded nearly $31,000, there was an ultimate balance on the right side which was added to endowments. With the hospital is associated the Newton District Nursing Association, which had 10,934 calls during the year.

